# Evaluation of basic life support practice skills based on artificial intelligence technology: system construction and evaluation equivalence validation

**DOI:** 10.1186/s12909-026-09285-6

**Published:** 2026-04-29

**Authors:** Yan Jiang, Yan Chen, Yin Zhang, Jiayu Wang, Min Tang, Yingchao Shi, Wenwen Qi, Yirong Sun, Jue Wang

**Affiliations:** 1 Nursing Department, Ruijin Hospital Shanghai Jiaotong University School of Medicine, Shanghai, 200025 China; 2 Nursing Department, Ruijin-Hainan Hospital Shanghai Jiaotong University School of Medicine (Hainan Boao Research Hospital), Qionghai, 571437 China; 3 Department of Medical Education, Ruijin Hospital Shanghai Jiaotong, University School of Medicine, Shanghai, 200025 China; 4 Department of Emergency, Ruijin Hospital Shanghai Jiaotong University School of Medicine, Shanghai, 200025 China; 5 Functional Neurosurgery, Ruijin Hospital Shanghai Jiaotong University School of Medicine, Shanghai, 200025 China

**Keywords:** Artificial intelligence, New nurses, Basic life support practice skills, Assessment, Equivalence verification

## Abstract

**Background:**

Basic life support (BLS) skills are the core competencies of healthcare professionals in responding to emergency situations. Traditional manual assessments suffer from subjective bias, low efficiency, and are affected by examiner fatigue. Artificial intelligence (AI) technology brings innovative opportunities for skill assessment, but currently there is a lack of mature and validated BLS AI assessment systems.

**Aim:**

To construct a basic life support practice skills evaluation system based on artificial intelligence technology, and verify its equivalence to manual evaluation. The purpose is to construct an evaluation system of basic life support practice skills based on artificial intelligence technology, verify its equivalence with manual evaluation, and analyse the advantages and development direction of artificial intelligence technology in the evaluation of basic life support practice skills.

**Methods:**

The BLS practical skills evaluation system based on AI technology was developed based on RTMPose, ST-GCN, SVM, YOLOX and Whisper AI base models, and the BLS practical skills assessment environment was constructed, which contains audio and video capture systems, CPR simulators with distance sensors, and displays for human–computer interaction with candidates. Using a paired design, the BLS practical skills assessment was conducted in August 2024 among 85 new nurses of the class of 2024 at Ruijin Hospital of Shanghai Jiaotong University School of Medicine, where each candidate's BLS skills performance was assessed by both the examiner and the AI system. The differences between the examiner scores and the AI system scores were compared, and at the same time, the self-assessment results of the Visual Analogue Scale of Fatigue Severity (VAS-F) of the four examiners before and after the scores were collected, as well as the candidates' satisfaction and acceptance of the application of the system for the BLS practical skills assessment.

**Results:**

There was no significant difference between AI system scores and examiner scores (t = -0.294, *p* = 0.769), with good absolute agreement further confirmed by an intraclass correlation coefficient of 0.868 (95% CI: 0.803–0.912). Examiner VAS-F self-ratings were 73.50 ± 19.33 before and 82.25 ± 25.10 after the examination, respectively, and 51 (64.56%) of the candidates indicated that the AI system assessment helped to reduce their nervousness.

**Conclusion:**

AI system marking is consistent with the results of examiner marking and can be used to evaluate BLS practical skills, and, the application of AI system can improve the effectiveness of the assessment organisation. Meanwhile, the candidates' acceptance of the AI system was good, and the application can be expanded after further optimisation of the system. Candidates showed good acceptance of the AI system; however, expansion of application should proceed only after further optimization of system stability to reduce failure rates below 5%.

## Introduction

Cardiac respiratory arrest (CRA) is an important health problem leading to death in adults. Early initiation of cardiopulmonary resuscitation (CPR), high-quality chest compressions, and rapid defibrillation, which are key steps of basic life support (BLS), are associated with improved patient prognosis [1]. In order to comprehensively and accurately evaluate a candidate's BLS skills, the evaluation should include the process of BLS and the initiation speed of CPR, the quality of CPR, the defibrillation process and the correct application of defibrillation equipment during BLS, which often challenges the examiner's observation, attention, and concentration, leading to high cognitive demands on the examiner, who, after being in such a state for a long period of time, is likely to suffer from cognitive fatigue, thus affecting the accuracy of their evaluation [2]. In recent years, the rapid development in the field of artificial intelligence (AI) technology may help to improve this situation. A number of studies have been conducted to explore the application of AI technology in the field of microsurgical skill evaluation [3], which integrated machine learning methods, computer vision and other technologies to achieve automatic evaluation of surgical performance. Introducing AI into BLS skills assessment involves core propositions of assessment theory. Based on Messick's validity framework, this study addresses three key issues: ⅰ. Construct validity: Does the AI system capture the essential construct of BLS competence, or merely recognize surface behavioral patterns? ⅱ. Consequential validity: What washback effects does AI assessment have on teaching and learning? ⅲ. Epistemological stance: Automated scoring reduces competence to quantifiable data streams, creating tension with 'clinical reasoning' emphasized in medical education. This study explicitly positions the AI system as an 'auxiliary tool for behavioral observation,' preserving space for human examiners' professional judgment in complex scenarios.This study develops a basic life support practice skills evaluation system based on artificial intelligence technology, demonstrates its feasibility by verifying its equivalence with the examiner's evaluation, and further analyses its practical application, proposing a future direction of exploration for the application of artificial intelligence technology in the field of clinical practice skills evaluation, which is now reported as follows.

## Data and methods

### Basic information

During August 2024, 95 newly recruited nurses from Ruijin Hospital of Shanghai Jiaotong University School of Medicine (hereinafter referred to as Ruijin Hospital) were included for the BLS practical skills assessment, which was scored by the AI-based BLS practical skills evaluation system (hereinafter referred to as the AI system) independently developed by the Ruijin Hospital, and at the same time an examiner was present in the examination room A total of 4 examiners participated in this study (all examiners included in this study had more than 5 years of experience in administering BLS practical skills assessment).

Candidates' inclusion criteria: ⅰ. Voluntary participation in this study and signing of informed consent; ⅱ. Commitment to comply with the study procedures and co-operate with the implementation of the whole process of the study. Exclusion Criteria:ⅰ. Being pregnant; ⅱ. Those whose physical condition does not allow them to complete the BLS operation; ⅲ. Those who are unable to complete the examination for various reasons. The study was approved by the Ethics Committee of Ruijin Hospital, Shanghai Jiaotong University School of Medicine (Ethics No. 2024 Linlun Audit No. 295), and all participants gave informed consent.

This study used convenience sampling, including all available newly recruited nurses at Ruijin Hospital in August 2024 (*n* = 95). Considering a 10% technical failure exclusion rate, 85 valid paired data were finally included. Post-hoc power analysis showed that under conditions of α = 0.05 (two-tailed), paired t-test, 85 pairs of samples could detect a minimum effect size of Cohen's d = 0.31 (small-to-medium effect) with 80% power; the actually observed effect size was d = 0.04 (based on AI-examiner score difference of −0.40 points, standard deviation 9.89), far below the detectable threshold, supporting the reliability of the "no significant difference" conclusion.

### Methods

#### Development of an AI-based BLS practice skills evaluation system

**Fig. 1:**
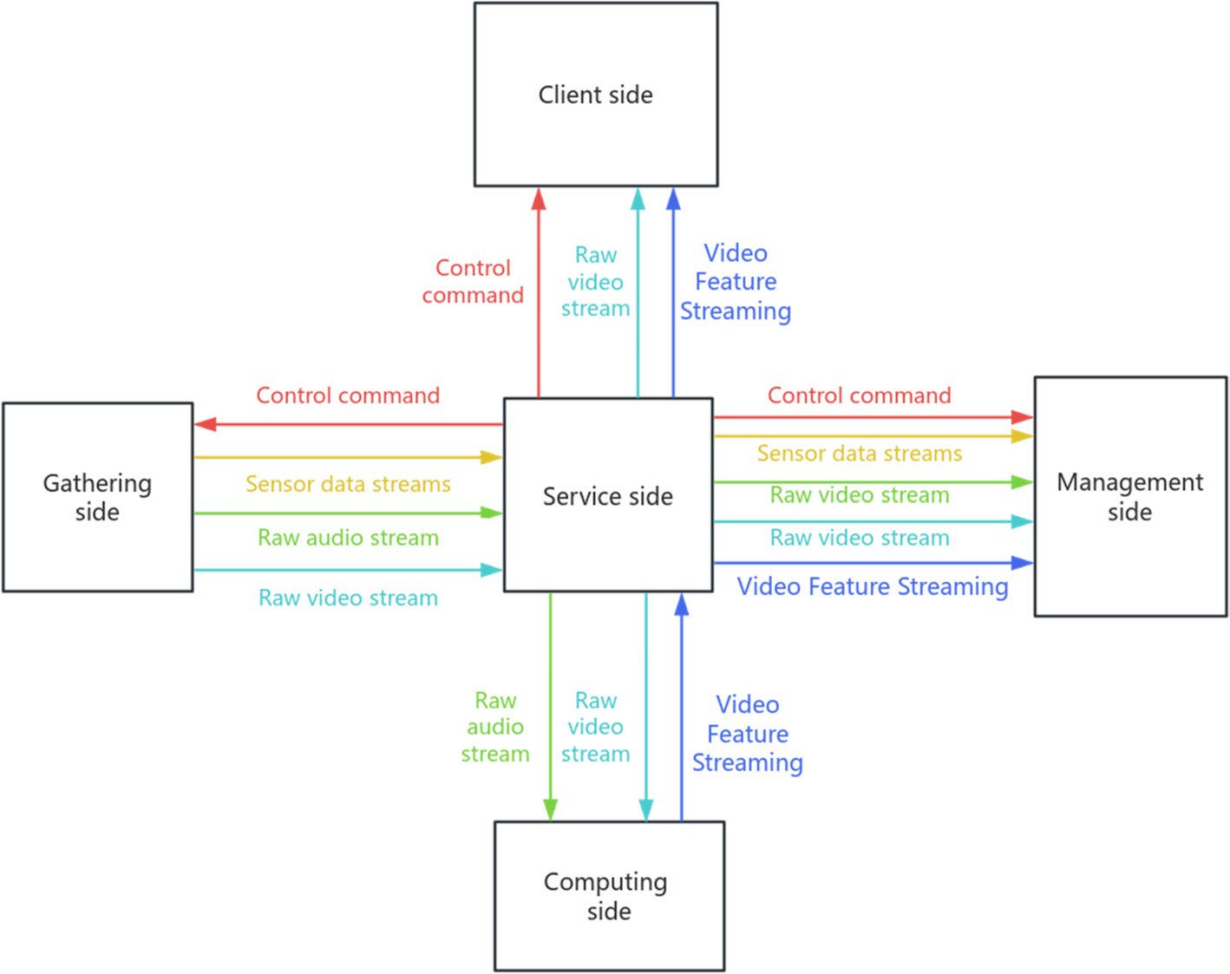
Schematic diagram of the link between the workstation of the AI-based BLS practical skills evaluation system and the various technical terminals

The AI system was applied to this study after repeated testing to confirm accuracy at the Medical Simulation Centre of Ruijin Hospital. The system was developed step by step based on several AI base models, applying the RTMPose model for human posture estimation, the ST-GCN model for action recognition, the SVM model for correction of video image recognition results, the YOLOX model for instrument recognition, and the Whisper model for audio recognition and semantic analysis, and integrating the above AI base models to develop this system. At the same time, the research team adapted the Ruijin Hospital Adult Single BLS Practical Skills Evaluation Scale for New Employees into a computer-processable process, designed a multimodal data acquisition scheme for the information to be determined in the process, including audio and video data and sensor data, and the data were transmitted in real time to the AI assessment workstation (server-side) to carry out comprehensive processing and arithmetic operations to achieve machine scoring (see Fig. 1). After that, the machine learning is completed in stages: the first stage through 342 video data for machine learning of BLS key action identification and determination; the second stage through 158 video data of the complete assessment process for machine learning of BLS process and determination of correctness of actions in the process.

In the machine learning stage, to enable the AI system to understand and process evaluation entries correctly, the evaluation tool was first transformed into a form that can be computationally processed by the computer [4]. The evaluation scoring form was set up with 50 entries, each scoring 0 or 2 points. The computer determines whether the candidate has completed the content of the corresponding entry by capturing the video image and audio image of the candidate, and gives the corresponding score.

For data acquisition, the feasibility, convenience, and operating costs were considered to design a multimodal data acquisition scheme [5]. The image data of candidates' behaviours are obtained through unobstructed video streams [6], and the audio streams are obtained using directional sound pickup devices, with speech recognition capabilities powered by large-scale self-supervised models such as Whisper [7] or wav2vec 2.0 [8], enabling accurate transcription of candidate verbal responses during the assessment. The distance sensors are used to accurately calculate the chest wall displacement of the simulators to determine CPR quality parameters. These data acquisition devices must not be in the candidate's path of travel and must not affect the candidate's tactile sensation to avoid interfering with the candidate's behaviours [9].

#### Construction of the assessment scene

The BLS practical skills assessment scene contains, i. A simulator capable of CPR operation: the simulator is equipped with a distance sensor under the chest wall, which is used for real-time sensing of the distance of the simulator's chest wall changes, in order to calculate the depth of the chest compression and the degree of chest resilience, as commonly implemented in advanced CPR simulators [10]. ⅱ. Multimedia monitor: equipped with AI system, camera and voice input function. iii. AI assessment workstation: receives the data transmitted by the above equipment and performs real-time calculation. iv. Other simulation equipment and consumables required to complete the BLS practical skills assessment: simulated external automated defibrillator, disposable respiratory filter membrane.

#### Examination implementation

**Fig. 2:**
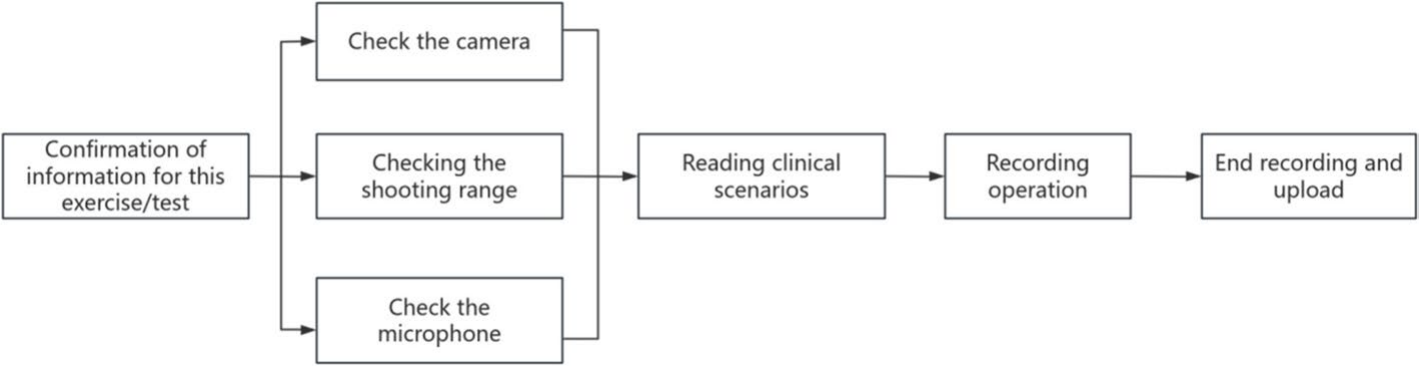
Flow chart of the assessment process of the AI-based BLS practical skills evaluation system

 During the examination process, after confirming the completion of self-preparation, the candidates check the status of the data acquisition equipment according to the voice prompts of the AI system, and after confirming that there is no error, they say ‘start the examination’ into the microphone, which is the entry into the process of the BLS practical skills assessment. 2 min after the candidates perform the first chest compressions, the voice of the AI system announces ‘AED has arrived’. The AI system will announce "AED arrives at the scene! The AI system voice broadcasts ‘end of examination’ after the candidate completes defibrillation and starts chest compressions again, and the AI system automatically scores the audio and video data collected throughout the examination, and the examination process of the AI system is shown in Fig. 2. During the examination process, an examiner observes the candidate's behaviour and scores the candidate's performance in real time through a one-way glass outside the examination room. During the examination process, an examiner observes the candidates' behaviour in real time through the one-way glass outside the examination room and scores them.

### Observation indicators

#### Candidates' BLS practical skills assessment scoring

The examiner and the AI visual determination system method all adopt the unified ‘Ruijin Hospital New Employee Adult Single BLS Practical Skills Evaluation Scale’, and the evaluation includes a number of key contents such as the assessment of the environment and the patient, the activation of the emergency response system, the quality of the CPR and the operation process of the AED, etc., and there are a total of 50 items in total, and the scoring method is checklist. There are 50 entries in total, using checklist scoring method, each entry gets 2 points if it is achieved, and 0 points if it is not achieved, with a full score of 100 points.

#### Examiner heart rate monitoring and fatigue self-assessment

Before the start and immediately after the end of the examination, the examiner's heart rate was measured by a finger pulse oximeter, and at the same time, the examiner was asked to complete the Visual Analogue Scale to Evaluate Fatigue Severity [VAF-S [11]]. The VAF-S consists of two subscales: fatigue severity (13 entries) and energy level (5 entries). Each entry describes a subjective feeling, and the answer is designed as a straight line with a length of 10 cm, and the two ends of the line represent the two extremes of this feeling: 0 (not at all) and 10 (extremely/very much), and the participant draws a circle on the number of the degree of severity according to the participant's own feeling, and the number circled is the score of the entry. The higher the VAF-S score, the more serious the fatigue.

#### Candidate satisfaction and acceptance survey

After the exam, a structured questionnaire survey was conducted among the candidates. This questionnaire was designed by the researchers themselves and includes the following content: i. Basic information: collect the name, age, gender and CPR training experience of the subjects. ⅱ. Candidate Satisfaction: Satisfaction with the overall environment of the AI system for the examination, comfort when using the AI system for the examination, and evaluation of the accuracy and reliability of the AI system. The questionnaire was evaluated using the Likert 5-point scale, with 5 points indicating very agreeable, 4 points indicating more agreeable, 3 points indicating generally agreeable, 2 points indicating less agreeable and 1 point indicating disagreeable. Candidate acceptance: Do you think the AI system in the CPR test helps reduce your sense of nervousness? The options are ‘Yes, it helps a lot’, ‘It helps to some extent’, ‘It doesn't help’, ‘It increases the sense of nervousness’. ".See the attachment.

### Statistical processing

Statistical analysis was performed using SPSS 27.0. Measurement data were expressed as mean ± standard deviation (± s), and count data as frequency and percentage (%). Quantitative data were analyzed using descriptive statistics. Corrected paired t-tests were used to compare differences between examiner and AI system scores (α = 0.05, two-tailed), and Cronbach's α coefficient was used to assess consistency reliability, with differences regarded as statistically significant at *p* < 0.05. To further evaluate the equivalence between AI system and examiner scores, the intraclass correlation coefficient (ICC) was calculated using a two-way random-effects model (absolute agreement, single rater; ICC(2,1)) with 95% confidence intervals. ICC values < 0.5, 0.5–0.75, 0.75–0.9, and > 0.9 were interpreted as poor, moderate, good, and excellent reliability, respectively [12]. Post-hoc power analysis was performed using G*Power 3.1.9.7, reporting effect size (Cohen's d) and achieved power (1-β).

## Results of the study

### General information of the study population

A total of 95 new nurses were included in this study. Among them, 23 (24.21%) were male and 72 (75.79%) were female; age (21.97 ± 1.38); 66 (69.47%) were specialists, 28 (29.47%) were undergraduates, and 1 (1.03%) was a master. The AI system was malfunctioned 10 times during the examination, including network delay, system lag, video not uploaded successfully, etc. Among them, 6 candidates re-examined the examination after the software engineers debugged the system on site, and another 4 candidates failed to complete the examination using the AI system due to the temporary malfunction that could not be lifted, but considering that the examination interruption may affect the psychological state of the candidates, which may affect their performance of the examination and their acceptance for the AI system, these 10 cases were accepted. However, considering that the interruption of the examination may affect the psychological state of the candidates, thus affecting their examination performance and acceptance of the AI system, the 10 cases were excluded, so the examination data of 85 candidates were finally included in this study.

### Appraisal scores

In this study, the BLS practical skills scores of 85 candidates were assessed by both the AI system and human examiners. The mean score assigned by the AI system was 81.95 ± 9.13, while the mean examiner score was 82.35 ± 8.65. A paired t-test revealed no statistically significant difference between the two scoring methods (t = −0.294, *p* = 0.769). The Cronbach's α coefficients were 0.693 for the AI system and 0.688 for the examiners, indicating comparable internal consistency. To further evaluate scoring equivalence, an intraclass correlation coefficient (ICC) was calculated using a two-way random-effects model (absolute agreement, single rater). The resulting ICC was 0.868 (95% CI: 0.803–0.912), indicating good absolute agreement according to predefined criteria. These findings are detailed in Tables 1 and 2.

**Table 1:** CPR skills assessment scores of new nurses [*n* = 85, (X ± s) points]

Grouping	Score	Cronbach's *α*
Human examiner marking	82.35 ± 8.65	0.688
AI system score	81.95 ± 9.13	0.693
*t*	0.294	
*p*	0.769

**Table 2:** Intraclass correlation coefficient between AI system and examiner scores (*n* = 85)

	Intraclass Correlation	95% Confidence Interval		F Test With True Value 0			
		Lower Bound	Upper Bound	Value	df1	df2	Sig
Single measures	0.868	0.803	0.912	14.113	84	84	0.000

Cronbach's α for AI system scoring was 0.693, and for examiner scoring was 0.688, both slightly below the conventional 0.70 threshold for acceptable internal consistency. However, the near-perfect equivalence of alpha values between AI and examiners (difference of only 0.005) supports that both methods demonstrate comparable measurement structure. This result actually aligns with the inherent characteristics of BLS assessment: this evaluation scale covers heterogeneous skills including scene safety judgment, compression technique, ventilation quality, and AED operation, rather than a single unidimensional construct; therefore, moderate internal consistency is reasonable. The traditional 0.70 threshold is more applicable to unidimensional psychometric instruments; for multidimensional procedural skill assessments, α values of 0.65–0.70 are acceptable.

### Self-assessment results of examiners' fatigue

There were no statistically significant differences in the self-assessment results of examiners' heart rate and fatigue before and after the examination. Heart rate showed no significant difference between pre- and post-examination measurements (77.50 ± 8.70 vs. 77.50 ± 12.40, t = 0.000, *p* = 1.000). Similarly, VAS-F scores demonstrated no significant difference (63.00 ± 29.99 vs. 76.25 ± 41.61, t = −0.867, *p* = 0.450) despite an observed trend toward increased fatigue.See Table 3 for details.

**Table 3:** Self-assessment results of examiners' heart rate and fatigue severity on the Visual Analogue Scale of Fatigue-Scales (VAF-S) before and after the examination (*n* = 4)

Item	Pre-evaluation	Post-evaluation	*t*	*p*	Cohen's *d*
Heart rate	77.50 ± 8.70	77.50 ± 12.40	0.000	1.000	0.000
VAS-F self-scoring	63.00 ± 29.99	76.25 ± 41.61	−0.867	0.450	0.434

### Results of candidate satisfaction and acceptance survey

An electronic questionnaire was distributed to 85 candidates and 79 were returned with a recovery rate of 92.94%. The Cronbach's alpha coefficient of the questionnaire was 0.842. 66 (83.54%) of them were satisfied with the overall environment of the assessment, 47 (59.49%) felt comfortable during the assessment, and 50 (63.29%) trusted the accuracy and reliability of the evaluation results of the AI system. 51 (64.56%) indicated that the AI system appraisal helped to reduce their sense of tension.The results of the satisfaction survey are detailed in Table 4.

**Table 4:** Candidates' satisfaction survey on AI system assessment [*n* = 79, (X ± s) score]

Item	Score
I am satisfied with the overall environment in which the AI system was used to conduct the BLS practical skills assessment	4.18 ± 0.69
I felt comfortable in using the AI system to conduct the assessment	3.87 ± 0.95
I recognise the accuracy and reliability of using the AI system in the BLS practical skills assessment	3.82 ± 0.76

## Discussion

### The difference between the AI system scoring and the examiner scoring is not statistically significant, and the AI system can be used to score the candidates' BLS practical skills

The t-test results of the AI system scoring and the examiner scoring show that the difference between the two is not statistically significant, suggesting that the result of the AI system scoring is the same as the examiner scoring, and that the scores obtained from the AI system can be used as the candidates' scores. To achieve the equivalence between the AI system and the examiner's scoring, this research team summarised the key steps in its development: ⅰ. In order to enable the AI system to understand and process the evaluation entries correctly, the developer needs to first transform the evaluation tool into a form that can be computationally processed by the computer [4]. In this study, the evaluation scoring form was set up with 50 entries, each scoring 0 or 2 points. The computer determines whether the candidate has completed the content of the corresponding entry by capturing the video image and audio image of the candidate, and gives the corresponding score. ⅱ. Accurate data acquisition in the real world is the basis for the AI system to produce accurate evaluation results, so the feasibility, convenience, and operating costs need to be considered when the system is developed to design a multimodal data acquisition scheme [5]. In this study, the image data of the candidates' behaviours are obtained through the capture of the unobstructed video streams [4], and the audio streams of the candidates are obtained by using directional sound pickup devices, with speech recognition capabilities powered by large-scale self-supervised models such as Whisper [7] or wav2vec 2.0 [8], enabling accurate transcription of candidate verbal responses during the assessment. The distance sensors are used to accurately calculate the chest wall displacement of the simulators to determine their CPR quality parameters. At the same time, these data acquisition devices must not be in the candidate's path of travel and must not affect the candidate's tactile sensation to avoid interfering with the candidate's behaviours [9]. ⅲ. For secondary development of AI models in the general domain, the machine learning stage requires not only a sufficient amount of data, but also data containing different levels of good and bad behavioural performance [13]. Under conditions where human examiners are prone to fatigue-induced fluctuations, AI systems can maintain consistency in scoring standards [14]. However these results apply only to standardized-trained new nurse populations (mean age 22 years, diploma/bachelor education, uniform BLS curriculum).

Notably, the Cronbach's α coefficients for AI system and examiner scoring were highly consistent (0.693 vs. 0.688). This 'equivalent reliability' phenomenon carries important methodological significance: first, it confirms that the AI system successfully replicated the measurement structure of human assessment rather than generating random error; second, the moderate alpha values of both reflect the multidimensional construct nature of BLS assessment itself—high-quality CPR requires integration of multiple relatively independent yet interrelated skill domains including environmental assessment, circulatory support, respiratory support, and defibrillation operation, rather than linear representation of a single ability. Therefore, pursuing excessively high internal consistency (e.g., α > 0.90) might instead indicate redundant items or overly narrow construct definition in the scale. Future studies could consider reporting subscale α coefficients (e.g., compression quality subscale, AED operation subscale) to more precisely assess measurement consistency of individual skill domains. Building on this evidence of structural equivalence, the absence of significant difference between AI and examiner scores (t = −0.294, *p* = 0.769) was further supported by a good intraclass correlation coefficient (ICC = 0.868, 95% CI: 0.803–0.912). This finding provides more robust evidence that the AI system's scoring is equivalent to that of human examiners, addressing a key methodological consideration in equivalence validation studies.

Furthermore, to address ethical governance requirements in high-stakes assessment, the AI system incorporates score explainability features that provide candidates with detailed feedback on specific performance dimensions (e.g., compression depth, ventilation volume, AED operation sequence). In cases of disputed results, a standardized accountability protocol is established: ⅰ. automated logging of all raw sensor data and video recordings for retrospective review; ⅱ. dual-blind reassessment by independent senior examiners; ⅲ. an appeals committee comprising clinical educators and technical specialists with authority to override AI-generated scores when systematic errors are identified; and ⅳ. continuous algorithm auditing to detect potential bias patterns.

### The AI system is free from cognitive fatigue concerns and is able to provide homogeneous evaluations for a centralised, high volume of candidates, enhancing the effectiveness of the assessment organisation

The results of the Examiner Fatigue Test in this study, although not statistically different, showed a trend of increasing VAF-S scores after the examination. Examiners will experience cognitive fatigue due to cognitive overload after performing long hours of highly focused evaluation work, which can seriously affect the evaluator's ability to effectively evaluate [2]. This phenomenon has been well documented in health professions education, where sustained high-cognitive-demand tasks impair rater-based assessment quality [15, 16]. The AI system shows obvious advantages in this regard, once the whole set of assessment scenarios start working normally, keeping the information transmission pathway open and the AI assessment workstation (server-side) able to perform normal computing, it can continuously implement the assessment and evaluation without stopping, which eliminates the need for the assessment organiser to arrange additional examiner breaks for assessments with a large number of candidates, shortening the total duration of the assessment. In addition, BLS practical skills evaluation requires examiners to observe and evaluate the key behaviours of candidates at high densities in a short period of time, and therefore often requires senior examiners with both many years of clinical experience and many years of experience in BLS teaching and evaluation to be able to perform the task; for the AI system, if the multimodal data collection scheme can be properly implemented during the pilot stage, standardised, multidimensional data collection can be achieved to achieve an accurate evaluation of each candidate's behaviour. Compared with traditional manual examiners, the AI system requires almost no additional examiners and saves a great deal of manpower costs. Therefore, in general the application of AI system can enhance the effectiveness of the assessment organisation.

Although no statistically significant difference was found in this study, examiner VAS-F scores increased from 73.50 ± 19.33 before the examination to 82.25 ± 25.10 after the examination, representing an increase of 8.75 points (approximately 11.9%). Given that only 4 examiners were included, this study was underpowered (power < 0.30, estimated based on medium effect size d = 0.5) to detect even moderate true differences. Effect size analysis revealed Cohen's d = 0.43, indicating a small-to-medium effect. This trend is consistent with literature reports: prolonged periods of highly focused evaluation work lead to cognitive overload and subsequent cognitive fatigue in examiners, seriously impairing their ability to evaluate effectively [17]. While the current data suggest that AI systems may help alleviate examiner fatigue, larger studies would be necessary to reach definitive conclusions.

### Candidates' acceptance of the AI system is good, and the application can be expanded after further optimising the system

The results of the satisfaction and acceptance survey of candidates in this study show that the application of the AI system for assessment and evaluation has a positive impact on the psychology of candidates, while there is potential room for improvement. The majority of candidates were satisfied with the current assessment scenario, the comfort level in the assessment, and suggested that the application of the AI system in the assessment might be able to reduce candidates' tension.

Candidates showed higher acceptance when interacting with the screen, and it is easier to accept this type of assessment as a generation growing up in a screen environment, which is supported by the sociological literature that screen interaction has a more positive impact on the younger generation [18]. At the same time, AI systems create a more equal assessment environment by eliminating the oppressive power of the "examiner's gaze" in traditional assessments [19]. However, the AI system experienced a total of 10 technical failures (10.5%) during the assessment process, including network delays, system lag, unsuccessful video uploads, and other system fluency and LAN transmission issues. This failure rate is unacceptable in high-stakes certification environments—most institutions require system availability exceeding 99% (i.e., failure rate < 1%). Technical failures lead to assessment interruptions, affecting candidates' psychological state and assessment continuity, potentially raising concerns about fairness, especially in high-stakes examinations [20]. This echoes the view in the literature that technology implementation may jeopardise the integrity of assessment data [21, 22]. To ensure clinical deployment feasibility of AI assessment systems, the following operational requirements must be met: ⅰ. Redundant network pathways: Establish primary and backup dual network channels with millisecond-level automatic failover; ⅱ. Local data caching: Configure local storage at data acquisition endpoints to ensure no data loss during network interruptions, with automatic resumption after recovery; ⅲ. Automated pre-examination system health checks: Mandate system health verification before assessments, including network latency testing, storage space validation, and sensor calibration; ⅳ. Standardized backup protocols: Develop examiner takeover protocols for technical failures, with clear criteria for failure determination and manual assessment switching procedures; ⅴ. Real-time monitoring and alerting: Deploy system performance monitoring platforms with threshold-based alarms for critical indicators including CPU load, memory usage, and network bandwidth. On the basis of optimising system stability, the scope and sample size of the study should be further expanded in the future to enhance the reliability and universality of the results.

## Research limitations

This study was a single-center study with a small sample size, focusing only on new nurses with 0–2 years of experience at our hospital; future research should expand the study population to verify the generalizability of our findings. However, a more fundamental limitation lies in the high context-specificity of AI model training and application: ⅰ. Population specificity: The system was trained exclusively on young new nurses with mean age 21.97 ± 1.38 years, not covering experienced nurses (who may demonstrate greater technique variability), physicians (with different compression depth/frequency habits), or emergency medical personnel; ⅱ. Scenario specificity: Assessments were conducted in a controlled simulation center using standardized single-rescuer BLS procedures, without involving the complexity of real clinical environments (noise interference, space constraints, family pressure) and time pressure; ⅲ. Equipment specificity: The system was calibrated based on specific brands of CPR manikins (with distance sensors) and AED trainers; different brands or models (e.g., Laerdal vs. Ambu manikins) may cause compression depth recognition bias due to chest wall stiffness and sensor precision differences; ⅳ. Technique specificity: Only adult single-rescuer CPR was covered, not including pediatric/neonatal resuscitation requiring modified techniques (e.g., thumb encircling method, 3:1 compression-to-ventilation ratio) or team-based resuscitation (role division, handover coordination); ⅴ. Protocol specificity: The assessment followed a fixed protocol (AED arrival at 2 min), not covering guideline variations across countries (AHA vs. ERC) or institution-specific protocols. Each of these new application scenarios would likely require retraining or transfer learning to maintain scoring accuracy. Future validation work should follow a "context-stratified validation" framework: first verifying cross-device/cross-population performance in simulated environments, then gradually transitioning to real clinical scenarios, and finally assessing cross-institution/cross-border generalizability.

This study had a small examiner sample size (*n* = 4), which limited the statistical power to test the effect of AI systems in reducing examiner fatigue. Future research should expand the examiner sample size to fully validate the potential advantages of AI systems in reducing assessor cognitive load.The 10.5% system failure rate in this study highlights the critical bottleneck of technical stability in clinical translation of AI assessment systems. Future studies validating AI assessment systems should report system uptime and reliability metrics as primary outcome indicators equally important to scoring validity. Based on the characteristics of AI technology, it is possible to further expand its application areas and develop AI-based training modules to provide learners with personalised feedback on practical skills evaluation to facilitate their targeted improvement of their practical skills.

In conclusion, although this study demonstrated acceptable equivalence between the AI system and human examiners under specific conditions (new nurses, single-center, simulated environment), the application of AI technology for BLS practical skills assessment in new nurses is feasible, credible, and can improve assessment organization effectiveness—opening a new pathway for medical education evaluation.

## Data Availability

The datasets generated and/or analyzed during the current study are not publicly available due to ethical restrictions and privacy concerns. De-identified aggregate scoring data (AI scores and examiner scores without candidate identifiers), anonymized questionnaire responses (with age, gender, and training experience retained but names removed), and system log files (timestamps, error codes, sensor readings) are available from the corresponding author upon reasonable request and with appropriate ethical approval. Video recordings of candidate performance cannot be shared due to identifiability concerns despite facial blurring, as body characteristics and voice may still permit recognition. AI model weights and training dataset remain proprietary intellectual property of Ruijin Hospital and are not available for external distribution.

## Abbreviations

AI: Artificial Intelligence
BLS: Basic Life Support
CPR: Cardiopulmonary Resuscitation
RTMPose: Real-Time Multi-Person Pose Estimation
ST-GCN: Spatial-Temporal Graph Convolutional Network
SVM: Support Vector Machine
YOLOX: You Only Look Once version X
Whisper: Automatic Speech Recognition Model by OpenAI
VAS-F: Visual Analogue Scale of Fatigue Severity
CRA: Cardiac Respiratory Arrest
AED: Automated External Defibrillator

## References

[1] Agarwal A, Baitha U, Ranjan P, et al. Knowledge and skills in cardiopulmonary resuscitation and effect of simulation training on it among healthcare workers in a tertiary care center in India. Indian J Crit Care Med. 2024;28(4):336–42. 10.5005/jp-journals-10071-24670. 38585308 10.5005/jp-journals-10071-24670 PMC10998517

[2] Paravattil B, Wilby KJ. Optimizing assessors’ mental workload in rater-based assessment: a critical narrative review. Perspect Med Educ. 2019;8(6):339–45. 10.1007/s40037-019-00535-6. 31728841 10.1007/s40037-019-00535-6 PMC6904389

[3] Bykanov AE, Danilov GV, Kostumov VV, et al. Artificial intelligence technologies in the microsurgical operating room (review). Sovrem Tekhnol Med. 2023;15(2):86–94. 10.17691/stm2023.15.2.08. 10.17691/stm2023.15.2.08 PMC10306972 37389018

[4] Liu Q, Jiang X, Jiang R. Classroom behavior recognition using computer vision: a systematic review. Sensors. 2025;25(2):373. 10.3390/s25020373. 39860742 10.3390/s25020373 PMC11769068

[5] Kaestner L. Artificial intelligence: training the trainer. Br J Haematol. 2022;198(5):805–6. 10.1111/bjh.18358. 35822904 10.1111/bjh.18358

[6] Zhan F, Yu Y, Wu R, et al. Multimodal image synthesis and editing: the generative AI era. IEEE Trans Pattern Anal Mach Intell. 2023;45(12):15098–119. 10.1109/TPAMI.2023.3305243. 37624713 10.1109/TPAMI.2023.3305243

[7] Radford A, Kim JW, Xu T, et al. Robust Speech Recognition via Large-Scale Weak Supervision. In: Proceedings of the 40th International Conference on Machine Learning (ICML). 2022. 10.48550/arXiv.2212.04356.

[8] Baevski A, Zhou Y, Mohamed A, Auli M. wav2vec 2.0: a framework for self-supervised learning of speech representations. Adv Neural Inform Process Syst (NeurIPS). 2020;33:12449–60. 10.48550/arXiv.2006.11477.

[9] Lipkova J, Chen RJ, Chen B, et al. Artificial intelligence for multimodal data integration in oncology. Cancer Cell. 2022;40(10):1095–110. 10.1016/j.ccell.2022.09.012. 36220072 10.1016/j.ccell.2022.09.012 PMC10655164

[10] Lee DK, Choi H, Jheon S, Jo YH, Im CW. Development of an extended reality simulator for basic life support training. IEEE J Transl Eng Health Med. 2022;10:4900507. 10.1109/JTEHM.2022.3152365. 35937462 10.1109/JTEHM.2022.3152365 PMC9342859

[11] Lee KA, Hicks G, Nino-Murcia G. Validity and reliability of a scale to assess fatigue. Psychiatry Res. 1991;36(3):291–8. 10.1016/0165-1781(91)90027-m. 2062970 10.1016/0165-1781(91)90027-m

[12] Koo TK, Li MY. A guideline of selecting and reporting intraclass correlation coefficients for reliability research. J Chiropr Med. 2016;15(2):155–63. 10.1016/j.jcm.2016.02.012. 27330520 10.1016/j.jcm.2016.02.012 PMC4913118

[13] Goodfellow I, Bengio Y, Courville A. Deep Learning. Cambridge, MA: MIT Press; 2016.

[14] Tekin M, Yurdal MO, Toraman Ç, Korkmaz G, Uysal İ. Is AI the future of evaluation in medical education? AI vs. human evaluation in objective structured clinical examination. BMC Med Educ. 2025;25:641. 10.1186/s12909-025-07241-4. 40312328 10.1186/s12909-025-07241-4 PMC12046780

[15] Tavares W, Ginsburg S, Eva KW. Selecting and simplifying: rater performance and behavior when considering multiple competencies. Teach Learn Med. 2016;28(1):41–51. 10.1080/10401334.2015.1107485. 26787084 10.1080/10401334.2015.1107489

[16] Naismith LM, Cavalcanti RB. Validity of cognitive load measures in simulation-based training: a systematic review. Acad Med. 2015;90(11):S24–35. 10.1097/ACM.0000000000000893. 26505098 10.1097/ACM.0000000000000893

[17] Weber LS, Schulze R. The effects of examiner fatigue on the diagnostic accuracy of dental radiographs. Clin Oral Investig. 2021;25(11):6193–9. 10.1007/s00784-021-03918-4. 10.1007/s00784-021-03918-4 33929630

[18] Lee S, Jirásek I. Associations between screen-based activity, spiritual well-being, and life satisfaction among adolescents. J Relig Health. 2019;58(3):795–804. 10.1007/s10943-017-0429-6. 28660472 10.1007/s10943-017-0429-6

[19] Brown C, Khavandi S, Sebastian A, et al. The influence of candidates’ race on examiners’ ratings in standardised assessments of clinical practice. Med Teach. 2025;47(3):492–7. 10.1080/0142159X.2024.2345266. 38771961 10.1080/0142159X.2024.2345266

[20] Curto G, Comim F. SAF: stakeholders’ agreement on fairness in the practice of machine learning development. Sci Eng Ethics. 2023;29(4):29. 10.1007/s11948-023-00448-y. 37486434 10.1007/s11948-023-00448-y PMC10366323

[21] Johnson MS. Responsible AI for measurement and learning: principles and practices. ETS Research Report RR-25-03. Princeton, NJ: Educational Testing Service; 2025.

[22] Becker B, van Rijn P, Molenaar D, Debeer D. Item order and speededness: implications for test fairness in higher educational high-stakes testing. Assess Eval High Educ. 2021;47(7):1030–42. 10.1080/02602938.2021.1991273.

